# Phylodynamic and Genetic Diversity of Canine Parvovirus Type 2c in Taiwan

**DOI:** 10.3390/ijms18122703

**Published:** 2017-12-13

**Authors:** Yung-Cheng Lin, Shu-Yun Chiang, Hung-Yi Wu, Jih-Hui Lin, Ming-Tang Chiou, Hsin-Fu Liu, Chao-Nan Lin

**Affiliations:** 1 Department of Medical Research, Mackay Memorial Hospital, Taipei 10449, Taiwan; lin65mt@gmail.com; 2 Department of Nursing, Shu-Zen Junior College of Medicine and Management, Kaohsiung 82144, Taiwan; 3 Department of Veterinary Medicine, College of Veterinary Medicine, National Pingtung University of Science and Technology, Pingtung 91201, Taiwan; chiang514040@hotmail.com; 4 Graduate Institute of Veterinary Pathobiology, College of Veterinary Medicine, National Chung-Hsing University, Taichung 40227, Taiwan; hwu2@dragon.nchu.edu.tw; 5 Center of Diagnostics and Vaccine Development, Centers for Disease Control, Taipei 11561, Taiwan; jeffy320@cdc.gov.tw; 6 Animal Disease Diagnostic Center, College of Veterinary Medicine, National Pingtung University of Science and Technology, Pingtung 91201, Taiwan; 7 Department of Bioscience and Biotechnology, National Taiwan Ocean University, Keelung 20224, Taiwan; 8 Department of Nursing, National Taipei University of Nursing and Health Sciences, Taipei 11219, Taiwan

**Keywords:** canine parvovirus 2c, Phylodynamic, Time to the most recent common ancestor

## Abstract

Canine parvovirus type 2c (CPV-2c) emerged in 2000 and is known for causing a more severe disease than other CPV-2 variants in puppies. In 2015, the emerging CPV-2c variant was isolated in Taiwan and it subsequently became the predominant variant. To trace the evolution of Taiwanese CPV-2c, we compared complete VP2 genes of CPV-2c from Taiwan and sequences obtained from GenBank. The evolutionary rate of CPV-2c was estimated to be 4.586 × 10^−4^ substitutions per site per year (95% highest posterior density (HPD) was 3.284–6.076 × 10^−4^). The time to the most recent common ancestor (TMRCA) dated to 1990 (95% HPD: 1984–1996) and 2011 (95% HPD: 2010–2013) for the CPV-2c variant and Taiwanese isolates, respectively. The CPV-2c variant isolated from Taiwan was clustered with CPV-2c from China. This phylogenetic clade began to branch off in approximately 2010 (95% HPD was 3.823–6.497). Notably, two unique mutations of Taiwanese CPV-2c were found, Q383R and P410L. In summary, this is the first report on the genome evolution of CPV-2c in Taiwan, revealing that this CPV-2c variant shares a common evolutionary origin with strains from China. The demographic history inferred by the Bayesian skyline plot showed that the effective population of CPV-2c increased until 2006 and then slowly declined until 2011.

## 1. Introduction

Canine parvovirus type 2 (CPV-2) is a non-enveloped single-stranded linear DNA virus belonging to the genus *Protoparvovirus*, a member of the *Parvoviridae* family. The virus has two major open reading frames, one encoding the nonstructural proteins (NS1 and NS2) and the other encoding the capsid proteins (VP1 and VP2) [1]. Based on antigenical and genetic differences, CPV-2 was divided into three variable types (CPV-2a, 2b and 2c). The critical difference of variability was observed in residue 426 of the VP2 protein. The position was asparagine (Asn) in 2a, aspartic acid (Asp) in 2b and glutamic acid (Glu) in 2c [2,3]. The functions of the capsid protein VP2 include providing receptor binding, controlling the host range [4,5,6] and eliciting neutralizing antibodies [3].

The original CPV-2 was recognized in the late 1970s. Subsequently, it was replaced as the predominant form in the dog population of the United States by CPV-2a in approximately 1980. CPV-2b and CPV-2c were first detected in 1984 and 2000 in the United States and Italy, respectively [7]. Although CPV-2c has similar clinical signs to CPV-2a and CPV-2b, it has been reported to be associated with a more severe disease [8,9].

The mean emerging date estimated for the CPV-2 clade from feline panleukopenia virus was 1968. After CPV-2 diverged into CPV-2 and CPV-2a, the mean age of CPV-2a was 8 years after CPV-2 first emerged [1]. However, little information on the mean age of CPV-2c was available to develop an age estimate. Another retrospective analysis revealed that the oldest CPV-2c strain was identified in 1996 in Germany [10]. A retrospective analysis revealed that the frequency of the CPV-2 variants in Italy underwent a rapid fluctuation during 1995–2005, with CPV-2c rapidly replacing CPV-2b [11]. To date, the CPV-2c variant has been predominant worldwide except for Asian countries [8,12,13,14,15,16,17,18,19,20,21,22,23].

The first case of the CPV-2c variant in Taiwan was reported in 2015 and clinical isolates of CPV-2c significantly increased to the level of 54.6% to become the dominant variant [18]. Moreover, the novel CPV-2c variant was found to be distributed throughout Taiwan [18]. Previously, CPV-2a and CPV-2b were the major antigenic variants in Taiwan during the past two decades [18,21,22,23]. The capsid protein region on CPV-2 is highly antigenic and is the target of many neutralizing antibodies. Therefore, to trace the evolutionary history of Taiwanese CPV-2c variants, we conducted a comprehensive phylogenetic and evolutionary analysis based on full-length VP2 sequences from five Taiwanese CPV-2c strains and reference sequences retrieved from GenBank.

## 2. Results

### 2.1. Sequence Comparison and Amino Acid Sequence Analysis

A total of 156 complete CPV-2c VP2 gene sequences including Taiwanese isolates and reference strains from the National Center for Biotechnology Information (NCBI) database (Supplementary Materials Table S1) were analyzed with MEGA software version 7 [24]. The result showed the amino acid distance was significantly low (1.50–0.23%) between our isolates and reference strains. Furthermore, several substitution sites were found at A5G, F267Y, Y324I, Q370R Q383R and P410L. These substitution positions have only been observed in Taiwanese and Chinese isolates. Notably, Q383R and P410L were determined to be two unique substitution positions in Taiwanese isolates (KU244254, KX421787) (Table 1).

### 2.2. Evolutionary Rates and the Most Recent Common Ancestor of CPV-2 and CPV-2c

**Figure 1:**
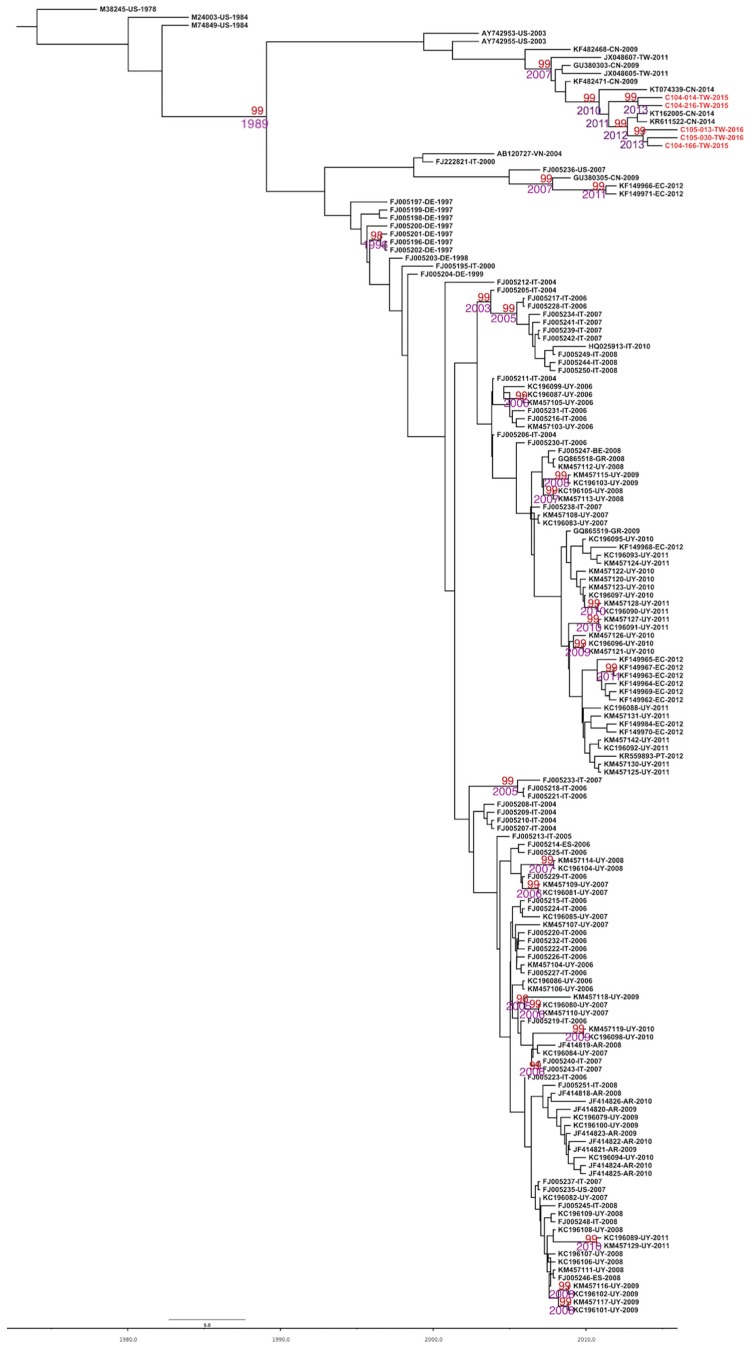
Maximum clade credibility (MCC) tree of CPV-2 inferred from 163 complete VP2 sequences. The MCC tree was constructed with 10% burn-in by Tree Annotator v 1.8 implemented in the BEAST software package. Red text represents the Taiwanese CPV-2c isolate in the present study. Numbers beside the branches are posterior probability values and branch time. Only posterior probability values above 0.95 are shown.

The dataset of CPV-2 and CPV-2c showed a positive correlation between genetic divergence and sampling time. Therefore, the dataset is suitable for phylogenetic molecular clock analysis in BEAST software. The uncorrelated exponential relaxed clock model and expansion growth population model were determined as the best fit models. The estimated nucleotide substitution rates of the VP2 region were 4.586 × 10^−4^ substitutions per site per year (95% highest posterior density (HPD) was 3.284–6.076 × 10^−4^) and 6.071 × 10^−4^ (95% HPD was 4.277–8.132) substitutions per site per year on CPV-2 and CPV-2c, respectively. The time to the most recent common ancestor (TMRCA) of CPV-2 was estimated to 1973 (95% HPD: 1963–1978). For the CPV-2c group, the overall TMRCA was dated to 1990 (95% HPD: 1984–1996) and to 2011 in Taiwanese isolates (95% HPD: 2010–2013). The evolutionary rate of the synonymous positions (third codon position) was 2.199, significantly higher than that of the nonsynonymous positions (first and second codon positions) (0.4) in the VP2 gene (Table 2). The strain C104-014/2015 (KU244254) was the first CPV-2c isolated in Taiwan and strains were clustered together with Chinese CPV-2c variants. The divergence of the Taiwanese lineage occurred in approximately 2010 (95% HPD was 2009–2012) (Figure 1).

### 2.3. Phylodynamics of CPV-2c

**Figure 2:**
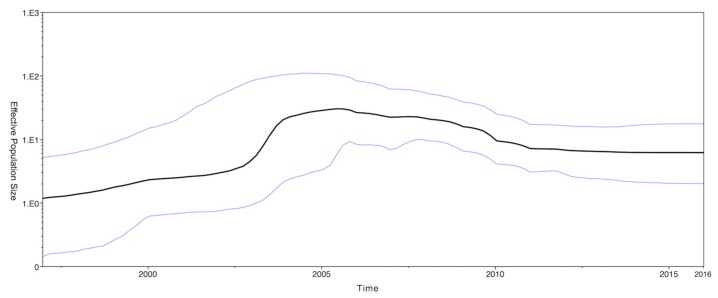
Bayesian skyline plot for the complete VP2 gene of the CPV-2. The *x*-axis is in units of year before 2016 and the *y*-axis represents the virus effective population size. The thicker bold line represents the median estimate of the effective number of infections over time and the thinner blue lines indicate the upper and lower bounds of the 95% HPD.

The phylodynamics of CPV-2c were estimated by a Bayesian skyline plot (BSP) based on the VP2 protein gene. The effective population size of CPV-2c was slowly rising until 2003, subsequently followed by a dramatic increase until 2005. After 2006, the effective population size decreased before remaining steady (Figure 2).

### 2.4. Selection Pressures in the CPV-2 VP2 Protein

The selection pressures of the VP2 protein in CPV-2 were estimated by the ratio of nonsynonymous substitutions (dN) to synonymous substitutions (dS). A dN/dS ratio of <1 represents negative selection; dN/dS ratio of 1 represents neutrality and dN/dS ratio of >1 represents positive selection. The overall mean dN/dS ratio in the CPV-2 VP2 gene was lower than 1 (0.108), implying that the gene was under negative selection. One positive selection was detected at codon 426 (Glu to Asn or Asp) using the FUBAR method (posterior probability was 0.97) (Table 3).

## 3. Discussion

CPV-2c is a new variant of CPV-2 that is now spreading worldwide. It causes gastroenteritis in puppy. The main clinical symptom among puppies in Taiwan is diarrhea or bloody diarrhea that also affects vaccinated dogs [18]. Studies have shown that both the CPV-2a and 2b variants constitute the prevalent CPV-2 field strains circulating in Taiwan in the past two decades [19,21,22,23]. However, this situation has since changed as the first case of CPV-2c was detected in 2015 and has since become the predominant strain (54.6%) in Taiwan [18]. 

Notably, a maximum likelihood (ML) tree revealed that CPV-2c isolated from Taiwan was clustered with Chinese strains, suggesting a closer phylogenetic relationship [18]. Results from a maximum clade credibility (MCC) tree (Figure 1) also support this observation, confirming that the recent CPV-2c isolate from Taiwan shares a common evolutionary origin with the Chinese CPV-2c and that the branching of that clade occurred in approximately 2010 (95% HPD was 3.823–6.497). Our results suggest that the Taiwanese isolate might have been imported from China and subsequently localized in Taiwan. 

Selection pressure estimation results reveal a dN/dS ratio of 0.108. This means that the VP2 protein gene was under purifying selection. A positive selection was found in position 426 on the capsid VP2 protein gene. The residue 426 was located in the top of loop 4 and has been defined as the major mutation site for CPV-2 variants. Such results are consistent with previous reports [25,26]. Our BSP result shows a steady state for the effective population size of CPV-2c in recent years (Figure 2). This implies that the genetic evolution of the VP2 gene for CPV-2 has stabilized. By dating the emergent time of CPV-2, we estimated the TMRCA to be in 1973 (95% HPD: 1968–1978). This result is in agreement with reports that CPV had been in the canine population for several years before it was first recognized in 1978 [1,26,27]. The CPV-2c clade has a mean age of 26 years, suggesting that it originated in 1990 (95% HPD: 1984–1996), approximately 10 years before the first discovery of CPV-2c in 2000 [7]. Indeed, a retrospective analysis revealed that the oldest CPV-2c strain was identified in 1996 in Germany [10]. Comparison of the substitution rates of CPV-2 and CPV-2c revealed that our results (4.586 × 10^−4^ and 6.071 × 10^−4^ substitutions per site per year) are slightly higher than those observed in Pereira’s (1.2 × 10^−4^ substitutions per site per year) [27], Shackelton’s (1.7 × 10^−4^ substitutions per site per year) [1] and Hoelzer’s (2.2 × 10^−4^ substitutions per site per year) [26] studies. This may be due to the differing amounts of CPV-2 strains analyzed (163 CPV-2 strains and 156 CPV-2c strains in the present study vs. 78, 56 and 90 CPV-2a strains in Pereira’s, Shackelton’s and Hoelzer’s studies, respectively). Nevertheless, our data agree that CPV-2 is a rapidly evolved DNA virus, particularly the CPV-2c variant (6.071 × 10^−4^ substitutions per site per year) and that its evolution rate has approached that of RNA viruses at approximately 10^−4^ substitutions per site per year [1,28].

VP2 encodes a viral capsid protein that is the major structural protein of CPV-2 and is involved in providing receptor binding, controlling the host range [4,5,6] and eliciting neutralizing antibodies [3]. Therefore, a few mutations may result in increased pathogenicity [29]. The recent Taiwanese CPV-2c strains showed several amino acid changes compared with prototype CPV-2c strains (FJ222821) such as A5G, F267Y, Y324I and Q370R in the VP2 protein. The mutation of F267Y has also been observed in Vietnam [30], China [31,32,33,34], India [35], Uruguay [36,37] and Portugal [16]. Residue 267 is not exposed on the capsid surface [38,39] and substitutions in this position may not affect the antigenicity of the virus. However, a previous study showed that the binding of DNA to the internal surface of the parvovirus protein shell inflicts specific conformational changes on the protein [38]. Therefore, the function of residue 267 remains to be elucidated. Y324I with two nucleotide changes (TAT→ATT) has been reported in China [31,32,33,34,40,41,42], Korea [43,44], Thailand [45], Japan [46], Taiwan [19,23], India [35,47], Uruguay [36,37] and Hungary [48]. Residue 324 of VP2 is subject to positive selection [26] and is adjacent to a residue (amino acid 323) known to be involved in host range and tropism via canine transferrin receptor binding [49]. Therefore, the function of residue 324 remains to be elucidated. The substitution of Q370R in the Taiwanese CPV-2c strain is within the VP2 protein and the mutation of Q370R was also observed in the giant panda in China [50] and Chinese CPV-2c strains [32,42]. Residue 370 is located between residues 359 and 375, which is a flexible surface loop of the capsid protein that is adjacent to a double Ca^2+^-binding site; these were found to be essential for virus infectivity. Changes in these residues are correlated with the ability of the virus to hemagglutinate erythrocytes [51]. Therefore, whether substitution of Q370R causes antigenic change remains to be investigated. In addition to the aforementioned mutations, A5G substitutions were identified in our CPV-2c and Chinese strain (KR611522, KT162005), which had not been detected before 2014. Therefore, further studies focusing on the potential variant CPV-2c strains should be conducted to elucidate the relationship between the A5G substitution and viral pathogenicity. Q383R and P410L are two unique substitutions sites in Taiwanese isolates. Residue 383 is not located in any loop. Therefore, that substitution may not affect the antigenicity of the virus. The amino acid 410 residue is located in loop 4, whose top protrusion site consists of residues 421–428 and 433–443, indicating that 410 is not in the top protrusion [39]. Whether these two unique substitution sites benefit the localization of the virus in Taiwan remains unclear, although Q383R and P410L would not change the polarity of amino acids and is not located in the top protrusion. Therefore, ongoing research on the functional effect of these two unique sites and continued monitoring of the gene evolution of CPV-2c are necessary.

## 4. Materials and Methods

### 4.1. Ethics Statement and Study Design

Ethical approval was obtained from the Institutional Animal Care and Use Committee of National Pingtung University of Science and Technology. This was a retrospective study without intervention or obtaining extra clinical specimens from dogs. A total of 163 complete CPV-2 VP2 sequences including Taiwanese isolates and reference strains from the NCBI database were used in this study, which contained 156 CPV-2c strains (Supplementary Materials Table S1).

### 4.2. Complete VP2 Gene Amplification and Sequencing

The antigenic type of the CPV-2 isolate was determined by partial VP2 gene PCR, as described by Buonavoglia et al. [7]. Five CPV-2c positive samples were amplified in two overlapping fragments of 1436 and 1031 bp to obtain the full length of the VP2 gene sequence by using two sets of newly designed and reference primers [7]: first set: forward, 5′-CGGTGCAGGACAAGTAAAA-3′ and reverse (Hrev), 5′-CATTTGGATAAACTGGTGGT-3′; second set: forward (Hfor), 5′-CAGGTGATG AATTTGCTACA-3′ and reverse, 5′-AATCTTAAAATAATRTGTAATAAAC-3′. The purified PCR products were cloned into the T & A^TM^ cloning Vector (Yeastern Biotech Co., Ltd., Taipei, Taiwan) and the presence of the desired insert in the recombinant plasmid DNA was confirmed by colony PCR with M13F and M13R universal primers. The target nucleotide sequences were determined in both orientations using an ABI automated sequencer (ABI 3730XL, Foster City, CA, USA). The viral nucleotide sequences determined in this study were deposited in GenBank with accession numbers KU244254 and KX421786-KX421789.

### 4.3. Phylodynamic Analysis

A full-length VP2 gene was aligned by T-coffee software [52]. The phylogenetic tree was constructed using the ML method in PhyML 3.0 (Available online: http://www.atgc-montpellier.fr/phyml/) [53]. Translation of the nucleotide sequences and estimation of the genetic distance were performed by MEGA software version 7 (Available online: http://www.megasoftware.net/) [24]. TempEst (TEMPoral Exploration of Sequences and Trees) was used to investigate the temporal signal [54]. The evolution rates of CPV-2 and CPV-2c were determined using the Bayesian Markov chain Monte Carlo (MCMC) method offered in BEAST v.1.8.4 (Available online: http://beast.bio.ed.ac.uk/) [55]. SRD06 was used as the best fit nucleotide substitution model because of its better resolution for coding regions for Bayesian analysis [56]. The demographic model, including constant size, expansion growth, Bayesian skyline and logistic growth, was used to estimate the evolutionary and population dynamics, under each as strict, lognormal relaxed and exponential relaxed molecular clock models [55]. The MCMC chains were run for a sufficient time to achieve convergence (Effective Sample Size > 200). The best fit demographic and clock model was estimated by Akaike’s information criterion (AICM) in the Tracer program v.1.6 [57]. An MCC tree was constructed by Tree Annotator v.1.8.4, with 10% burn-in. The final phylogenetic trees were edited by Figtree v.1.4.2. The accession numbers of the sequences used for evolutionary analysis in this study are listed in Supplementary Table S1.

### 4.4. Selection Pressure of CPV2 VP2 Protein Genes

To determine the selection pressures on the VP2 protein, we estimated the dN/dS ratio per site based on the maximum likelihood (ML) trees under the appropriate substitution model, using single-likelihood ancestor counting and fixed-effects likelihood methods, with the significance level set at 0.05. The Bayesian test for selection acting on individual sites was conducted using FUBAR, with the posterior probabilities set at 0.95 [58]. All the methods were implemented in the Datamonkey web server interface (http://www.datamonkey.org) [59,60].

## 5. Conclusions

This is the first report of CPV-2c genome evolution in Taiwan. Our results indicate that the recent CPV-2c isolate from Taiwan shares a common evolutionary origin with Chinese CPV-2c strains of and that a split of the Taiwanese lineage occurred in approximately 2010. The demographic history inferred from the BSP showed that the effective population of CPV-2c increased until 2006 and then slowly declined until 2011.

## Figures and Tables

**Table 1:** Characteristics of the Amino Acid Substitution of CPV-2c Worldwide.

Country	Year	Amino Acid at Position							Accession Numbers
		5	267	324	370	383	410	440	
Taiwan	2015	A	**Y ^a^**	**I ^a^**	**R ^a^**	**R ^a^**	**L ^a^**	T	KU244254
	2015	A	**Y ^a^**	**I ^a^**	**R ^a^**	**R ^a^**	P	T	KX421787
	2015	**G ^a^**	**Y ^a^**	**I ^a^**	**R ^a^**	Q	P	T	KX421786
	2016	**G ^a^**	**Y ^a^**	**I ^a^**	**R ^a^**	Q	P	T	KX421788, KX421789
China	2009	A	**Y**	**I**	Q	Q	P	T	GU380303
	2009	A	F	Y	Q	Q	P	T	GU380305
	2014	**G**	**Y**	**I**	**R**	Q	P	T	KR611522, KT162005
Argentina	2008–2009	A	F	Y	Q	Q	P	T	JF414818–JF414820
	2009–2010	A	F	Y	Q	Q	P	**A**	JF414821–JF414825
	2010	A	F	Y	Q	Q	P	T	JF414826
Belgium	2008	A	F	Y	Q	Q	P	T	FJ005247
Ecuador	2012	A	F	Y	Q	Q	P	T	KF149962–KF149965, KF149967–KF149971, KF149984,
	2012	A	F	Y	Q	Q	P	**S**	KF149966
Germany	1997–1999	A	F	Y	Q	Q	P	T	FJ005196–FJ005204
Greece	2008–2009	A	F	Y	Q	Q	P	T	GQ865518, GQ865519
Italy	2000–2010	A	F	Y	Q	Q	P	T	FJ222821, FJ005195, FJ005205–FJ005213, FJ005215–FJ005234, FJ005237–FJ005245, FJ005248–FJ005251, HQ025913
India	2014	A	F	Y	Q	Q	P	T	KP071956
Spain	2006–2008	A	F	Y	Q	Q	P	T	FJ005214, FJ005246
Uruguay	2006–2011	A	F	Y	Q	Q	P	T	KC196079–KC196093, KC196080–KC196093, KC196095–KC196109, KC196111–KC196114, KM457103–KM457110, KM457115–KM457131, KM457142
	2010	A	F	Y	Q	Q	P	**A**	KC196094
USA	2007	A	F	Y	Q	Q	P	T	FJ005235
	2007	A	F	Y	Q	Q	P	**A**	FJ005236
Vietnam	2004	A	F	Y	Q	Q	P	T	AB120727

**^a^** Bold represents the amino acid differing from the prototype CPV-2c strain (FJ222821).

**Table 2:** Mean Relative Evolutionary Rates for Codon Positions and TMRCA in VP2 gene.

	TMRCA	Substitution Rates Sub/Site/Year (10^−4^)	Mean Relative Substitution Rate	SE of Mean
VP2 gene		4.586 (3.284~6.076)		
CPV-2	1973 (1963–1978) ^a^			
CPV-2c	1990 (1984–1996)	6.071 (4.277~8.132)		
CPV-2c Taiwanese strains	2011 (2010–2013)			
1st + 2nd codon position			0.4 (0.302~0.495)	5.215 × 10^−4^
3rd codon position			2.199 (2.011~2.396)	1.043 × 10^−3^

^a^ ( ) Lower and upper 95% of highest posterior density (HPD).

**Table 3:** Selection Pressure Detected in CPV-2 VP2 Protein Gene.

	Positively Selected Sites			No. of Negatively Selected Sites			Mean dN/dS
	SLAC ^a^	FEL ^a^	FUBA ^b^	SLAC ^a^	FEL ^a^	FUBAR ^b^	
CPV-2 VP2 gene	Non	Non	426	8	46	31	0.108

^a^ *p* value of <0.05. ^b^ Posterior probability of ≥0.95.

## Supplementary Materials

Supplementary materials can be found at www.mdpi.com/1422-0067/18/12/2703/s1.
