# Supplementary material for: Phylodynamic and Genetic Diversity of Canine Parvovirus Type 2c in Taiwan
Source: Int J Mol Sci. 2017 Dec 13;18(12):2703. doi: 10.3390/ijms18122703 (PMC5751304; doi:10.3390/ijms18122703)
Supplement: Supplementary file 1 [file ijms-18-02703-s001.pdf]

Supplementary Table 1 Details of 163 complete VP2 genes of CPV-2 from GenBank.

Isolate	Year	Origin	Genotype	Accession No.
This study				
C104-014	2015	Taiwan (TW)	2c	KU244254
C104-166	2015	Taiwan (TW)	2c	KX421786
C104-216	2015	Taiwan (TW)	2c	KX421787
C105-013	2016	Taiwan (TW)	2c	KX421788
C105-030	2016	Taiwan (TW)	2c	KX421789
Reference				
CPV-42	2011	Taiwan (TW)	2a	JX048605
CPV-88	2011	Taiwan (TW)	2b	JX048607
Arg32	2008	Argentina (AR)	2c	JF414818
Arg35	2008	Argentina (AR)	2c	JF414819
Arg44	2009	Argentina (AR)	2c	JF414820
Arg48	2009	Argentina (AR)	2c	JF414821
Arg60	2009	Argentina (AR)	2c	JF414823
Arg64	2010	Argentina (AR)	2c	JF414822
Arg66	2010	Argentina (AR)	2c	JF414824
Arg67	2010	Argentina (AR)	2c	JF414825
Arg68	2010	Argentina (AR)	2c	JF414826
195/08	2008	Belgium (BE)	2c	FJ005247
06/09	2009	China (CN)	2c	GU380303
08/09	2009	China (CN)	2c	GU380305
CPV-SD-14-12	2014	China (CN)	2c	KR611522
HRB-A6	2014	China (CN)	2c	KT074339
G1	2009	China (CN)	2c	KF482468
G15	2009	China (CN)	2c	KF482471
BJ14-8	2014	China (CN)	2c	KT162005
2c_ME1_ECU2012	2012	Ecuador (EC)	2c	KF149962
2c_ME10_ECU2012	2012	Ecuador (EC)	2c	KF149963
2c_ME23_ECU2012	2012	Ecuador (EC)	2c	KF149964
2c_ME25_ECU2012	2012	Ecuador (EC)	2c	KF149965
2c_ME27_ECU2012	2012	Ecuador (EC)	2c	KF149967
2c_ME28_ECU2012	2012	Ecuador (EC)	2c	KF149984
2c_ME29_ECU2012	2012	Ecuador (EC)	2c	KF149968
2c_ME31_ECU2012	2012	Ecuador (EC)	2c	KF149969
2c_ME32_ECU2012	2012	Ecuador (EC)	2c	KF149971
2c_ME34_ECU2012	2012	Ecuador (EC)	2c	KF149970

2c_ME26_ECU2012	2012	Ecuador (EC)	2c	KF149966
G7/97	1997	Germany (DE)	2c	FJ005196
G51/97	1997	Germany (DE)	2c	FJ005197
G133/97	1997	Germany (DE)	2c	FJ005198
G172/97	1997	Germany (DE)	2c	FJ005199
G359/97	1997	Germany (DE)	2c	FJ005200
G362/97	1997	Germany (DE)	2c	FJ005201
G367/97	1997	Germany (DE)	2c	FJ005202
G52-9/2-98	1998	Germany (DE)	2c	FJ005203
G333/99	1999	Germany (DE)	2c	FJ005204
GR51/08	2008	Greece (GR)	2c	GQ865518
GR09/09	2009	Greece (GR)	2c	GQ865519
56/00	2000	Italy (IT)	2c	FJ222821
136/00	2000	Italy (IT)	2c	FJ005195
279/04	2004	Italy (IT)	2c	FJ005205
287/04	2004	Italy (IT)	2c	FJ005206
290/04	2004	Italy (IT)	2c	FJ005207
291/04	2004	Italy (IT)	2c	FJ005208
303/04	2004	Italy (IT)	2c	FJ005209
307/04	2004	Italy (IT)	2c	FJ005210
342/04	2004	Italy (IT)	2c	FJ005211
349/04	2004	Italy (IT)	2c	FJ005212
9/05	2005	Italy (IT)	2c	FJ005213
252/06	2006	Italy (IT)	2c	FJ005215
284/06	2006	Italy (IT)	2c	FJ005216
327/06	2006	Italy (IT)	2c	FJ005217
330/06	2006	Italy (IT)	2c	FJ005218
336/06	2006	Italy (IT)	2c	FJ005219
337/06	2006	Italy (IT)	2c	FJ005220
340/06	2006	Italy (IT)	2c	FJ005221
359/06	2006	Italy (IT)	2c	FJ005222
365/06	2006	Italy (IT)	2c	FJ005223
367/06	2006	Italy (IT)	2c	FJ005224
382/06	2006	Italy (IT)	2c	FJ005225
383/06	2006	Italy (IT)	2c	FJ005226
389/06	2006	Italy (IT)	2c	FJ005227
393/06	2006	Italy (IT)	2c	FJ005228
397/06	2006	Italy (IT)	2c	FJ005229

398/06	2006	Italy (IT)	2c	FJ005230
406/06	2006	Italy (IT)	2c	FJ005231
411/06	2006	Italy (IT)	2c	FJ005232
40/07	2007	Italy (IT)	2c	FJ005233
43/03	2007	Italy (IT)	2c	FJ005234
159/07	2007	Italy (IT)	2c	FJ005237
158/07	2007	Italy (IT)	2c	FJ005238
165/07-A	2007	Italy (IT)	2c	FJ005239
208/07	2007	Italy (IT)	2c	FJ005240
215/07-2	2007	Italy (IT)	2c	FJ005241
217/07	2007	Italy (IT)	2c	FJ005242
243/07	2007	Italy (IT)	2c	FJ005243
127/08-A	2008	Italy (IT)	2c	FJ005244
127/08-B	2008	Italy (IT)	2c	FJ005245
219/08-2	2008	Italy (IT)	2c	FJ005248
219/08-5	2008	Italy (IT)	2c	FJ005249
219/08-13	2008	Italy (IT)	2c	FJ005250
239/08	2008	Italy (IT)	2c	FJ005251
cat300/10	2010	Italy (IT)	2c	HQ025913
67/06	2006	Spain (ES)	2c	FJ005214
128/08	2008	Spain (ES)	2c	FJ005246
M181	2009	Uruguay (UY)	2c	KC196079
M101	2007	Uruguay (UY)	2c	KC196080
M95	2007	Uruguay (UY)	2c	KC196081
M86	2007	Uruguay (UY)	2c	KC196082
M82	2007	Uruguay (UY)	2c	KC196083
M72	2007	Uruguay (UY)	2c	KC196084
M57	2007	Uruguay (UY)	2c	KC196085
M55	2006	Uruguay (UY)	2c	KC196086
M52	2006	Uruguay (UY)	2c	KC196087
M354	2011	Uruguay (UY)	2c	KC196088
M349	2011	Uruguay (UY)	2c	KC196089
M346	2011	Uruguay (UY)	2c	KC196090
M326	2011	Uruguay (UY)	2c	KC196091
M317	2011	Uruguay (UY)	2c	KC196092
M307	2011	Uruguay (UY)	2c	KC196093
M269	2010	Uruguay (UY)	2c	KC196094
M258	2010	Uruguay (UY)	2c	KC196095

M247	2010	Uruguay (UY)	2c	KC196096
M242	2010	Uruguay (UY)	2c	KC196097
M235	2010	Uruguay (UY)	2c	KC196098
M21	2006	Uruguay (UY)	2c	KC196099
M196	2009	Uruguay (UY)	2c	KC196100
M187	2009	Uruguay (UY)	2c	KC196101
M185	2009	Uruguay (UY)	2c	KC196102
M173	2009	Uruguay (UY)	2c	KC196103
M169	2008	Uruguay (UY)	2c	KC196104
M152	2008	Uruguay (UY)	2c	KC196105
M135	2008	Uruguay (UY)	2c	KC196106
M129	2008	Uruguay (UY)	2c	KC196107
M104	2008	Uruguay (UY)	2c	KC196108
M120	2008	Uruguay (UY)	2c	KC196109
UY12	2006	Uruguay (UY)	2c	KM457103
UY47	2006	Uruguay (UY)	2c	KM457104
UY52	2006	Uruguay (UY)	2c	KM457105
UY55	2006	Uruguay (UY)	2c	KM457106
UY72	2007	Uruguay (UY)	2c	KM457107
UY82	2007	Uruguay (UY)	2c	KM457108
UY95	2007	Uruguay (UY)	2c	KM457109
UY101	2007	Uruguay (UY)	2c	KM457110
UY120	2008	Uruguay (UY)	2c	KM457111
UY135	2008	Uruguay (UY)	2c	KM457112
UY152	2008	Uruguay (UY)	2c	KM457113
UY169	2008	Uruguay (UY)	2c	KM457114
UY173	2009	Uruguay (UY)	2c	KM457115
UY185	2009	Uruguay (UY)	2c	KM457116
UY187	2009	Uruguay (UY)	2c	KM457117
UY190	2009	Uruguay (UY)	2c	KM457118
UY235	2010	Uruguay (UY)	2c	KM457119
UY242	2010	Uruguay (UY)	2c	KM457120
UY247	2010	Uruguay (UY)	2c	KM457121
UY258	2010	Uruguay (UY)	2c	KM457122
UY261	2010	Uruguay (UY)	2c	KM457123
UY307	2011	Uruguay (UY)	2c	KM457124
UY317	2011	Uruguay (UY)	2c	KM457125
UY318	2010	Uruguay (UY)	2c	KM457126

UY326	2011	Uruguay (UY)	2c	KM457127
UY346	2011	Uruguay (UY)	2c	KM457128
UY349	2011	Uruguay (UY)	2c	KM457129
UY354	2011	Uruguay (UY)	2c	KM457130
UY368	2011	Uruguay (UY)	2c	KM457131
UY370c	2011	Uruguay (UY)	2c	KM457142
HNI-4-1	2004	Vietnam (VN)	2c	AB120727
67/07-11	2007	USA (US)	2c	FJ005235
110/07-27	2007	USA (US)	2c	FJ005236
isolate 39	1984	USA (US)	2b	M74849
CPV-b	1978	USA (US)	2	M38245
CPV-15	1984	USA (US)	2	M24003
CPV-435	2003	USA (US)	2a	AY742953
CPV-436	2003	USA (US)	2b	AY742955
PT178/12	2012	Portugal (PT)	2c	KR559893
